# PTBP1 is a Novel Poor Prognostic Factor for Glioma

**DOI:** 10.1155/2022/7590997

**Published:** 2022-03-08

**Authors:** Pan Liu, Guo-Chao He, Yu-Zhen Tan, Ge-Xin Liu, An-Min Liu, Xiao-Peng Zhu, Yang Zhou, Wan-Ming Hu

**Affiliations:** ^1^Department of Emergency, The Affiliated Zhuzhou Hospital of Xiangya Medical College CSU, Zhuzhou 412007, China; ^2^Department of Trauma Center, The Affiliated Zhuzhou Hospital of Xiangya Medical College CSU, Zhuzhou 412007, China; ^3^Department of Neurosurgery, The Affiliated Zhuzhou Hospital of Xiangya Medical College CSU, Zhuzhou 412007, China; ^4^Department of Pathology, Sun Yat-sen University Cancer Center, State Key Laboratory of Oncology in South China, Collaborative Innovation Center for Cancer Medicine, Guangzhou 510060, China

## Abstract

**Objective:**

Polypyrimidine tract-binding protein 1 (PTBP1) is an RNA-binding protein, which plays a role in pre-mRNA splicing and in the regulation of alternative splicing events. However, little was known about the correlation between PTBP1 and glioma and its prognostic significance in glioma patients. Our aim was to investigate the expression, functional role, and prognostic value of PTBP1 in glioma.

**Methods:**

We explored the expression of PTBP1 protein using immunohistochemistry in 150 adult malignant glioma tissues and 20 normal brain tissues and evaluated its association with clinicopathological parameters by chi-square test. Kaplan-Meier method was used to evaluate the prognostic effect of PTBP1 in glioma. Univariate/multivariate Cox analyses were used to identify independent prognostic factors. Transcriptional regulation network was constructed based on differentially expressed genes (DEGs) of PTBP1 from TCGA/CGGA database. GO and KEGG enrichment analyses were used to explore the function and pathways of DEGs.

**Results:**

Out of the 150 malignant glioma tissues (60 LGG and 90 GBMs) and 20 normal brain tissues in our cohort, PTBP1 protein was high expressed in glioma tissues (79/150, 52.7%), but no expression was detected in normal brain tissues (0/20, 0%). The expression of PTBP1 was significantly higher in GBMs (*P* < 0.001). More than half of GBMs (62/90, 68.9%) were PTBP1 high expression. Chi-square test showed that the expression of PTBP1 was correlated with patient age, WHO grade, Ki-67 index, and IDH status. High expression of PTBP1 was significantly associated with poor prognosis in glioma, and it was an independent risk factor in glioma patients. Furthermore, we shed light on the underlying mechanism of PTBP1 by constructing a miR-218-TCF3-PTBP1 transcriptional network in glioma.

**Conclusion:**

PTBP1 was high expressed in glioma, and it significantly correlated with poor prognosis, suggesting a potential therapeutic target for glioma, particularly for GBM.

## 1. Introduction

Gliomas are the most common primary intracranial neoplasms, categorized into grades 1 to 4 according to the World Health Organization (WHO) grading system. In fact, except for pilocytic astrocytoma (WHO grade 1, a borderline tumor with unique molecular profiles and an extremely good prognosis), all the WHO 2-4 gliomas are malignant tumors. They are the most lethal brain tumors with the high morbidity and mortality rates. Despite the basic research in-depth and advances in surgical techniques over the past few years, the pathogenic mechanism of these malignant diseases remains unclear and the prognosis of them still has not been greatly improved. Currently, more and more researches are focused on the genetic aberrations to explore the deep-seated mysteries of these diseases and novel therapeutic strategies.

Among them, there is a growing consensus that error changes in RNA splicing play an important role in tumorigenesis. At present, the researchers have discovered that there are approximately 145 genes involved in RNA splicing, which are widely expressed in brain, breast, colon, and prostate cancer [1]. Polypyrimidine tract binding protein (PTB) is a multifunctional component of mRNA metabolism that affects alternative splicing, mRNA stability, polyadenylation, internal ribosome entry site-independent translation, and possibly transcription [2]. Several pieces of evidence support the role of PTBP1 in the development and progression of gliomas. Due to its role in RNA processing and nucleolar function, PTBP1 shows predominantly nuclear localization in tissues. It plays an important role in pre-mRNA splicing and in the regulation of alternative splicing events.

In this study, we analyzed the expression, prognostic value, and potential mechanism of PTBP1 by immunohistochemistry in a single-center cohort of 150 glioma patients and by a comprehensive bioinformatics analysis of TCGA and CGGA databases. Our findings revealed that PTBP1 was highly expressed in glioma, especially in GBM, and associated with pathological grade and predicted poor prognosis of patients, representing a potential therapeutic target.

## 2. Materials and Methods

### 2.1. Patients and Samples

Paraffin-embedded tissues were obtained from the archives of the department of pathology of Zhuzhou Central Hospital and Sun Yat-sen University Cancer Center, between 2015 and 2020. Written informed consent was acquired in all cases, and the protocols of this research were approved by the Scientific Ethics Committee of Zhuzhou Central Hospital and Sun Yat-sen University Cancer Center. The final samples were from 150 patients with gliomas (WHO II-IV), including 30 cases of WHO II (oligodendroglioma and astrocytoma), 30 cases of WHO III (anaplastic oligodendroglioma and astrocytoma), and 90 cases of WHO IV (glioblastoma) and 20 normal brain tissues from patients who received brain surgery for reasons other than glioma. Overall survival represents the time interval between the date of diagnosis and the date of death or the last known follow-up.

### 2.2. Immunohistochemistry (IHC) Assay

Formalin-fixed and paraffin-embedded (FFPE) glioma tissues were cut into 3-4 *μ*m sections, then deparaffinised using xylene, and hydrated through graded alcohol. Perform heat mediated antigen retrieval with citrate buffer (pH = 6.0) before commencing with IHC staining protocol. In short, the sections were incubated with PTBP1 antibody (ab133734, Abcam, USA) overnight at 4°C. Then, the secondary antibody (Dako, Denmark) was applied and incubated for 1 h at room temperature, followed by 3,3-diaminobenzidine tetra hydrochloride staining, and observed under microscope (BX51, Olympus, Japan). The expression of PTBP1 was calculated as the sum of the percent positivity of stained tumor cells and the staining intensity. The percent positivity was scored as follows: 1 for 0-25%, 2 for 26%-50%, 3 for 51%-75%, and 4 for >75%. The staining intensity was scored as follows: 0 for no staining, 1 for light yellow, 2 for yellowish brown, and 3 for dark brown. A final staining score of ≥6 was defined as high expression. All the samples were scored separately by three independent pathologists, who were blinded to the clinical data.

### 2.3. Bioinformatics Analysis

The RNA-sequencing data and patients' survival of PTBP1 in gliomas based on The Cancer Genome Atlas (TCGA) and Chinese Glioma Genome Atlas (CGGA) were analyzed with the GEPIA tool (http://gepia.cancer-pku.cn/). The degree of PTBP1 mRNA expression of glioblastoma and normal tissue was validated by using the Oncomine database (https://www.oncomine.org/resource/login.html). MicroRNAs were predicted using miRanda and Targetscan databases. Transcription factors (TFs) of PTBP1 were predicted using the GCBI database (https://www.gcbi.com.cn). In addition, the Gene Ontology (GO) enrichment analysis for biological process (BP) and Genomes (KEGG) pathway were analyzed by DAVID (https://david-d.ncifcrf.gov/).

### 2.4. Statistical Analysis

All the data were analyzed using the SPSS18.0 software (IBM, USA). *T*-test was applied for the comparison between two groups. The association between PTBP1 and clinicopathological parameters was assessed using 2 × 2 contingency tables and the chi-square (*χ*^2^) test. Kaplan-Meier survival curves, log-rank test, and multivariate Cox regression analyses were generated to estimate overall survival. Correlation analysis of PTBP1 was assessed by Pearson correlation method. *P* < 0.05 was defined statistically significant.

## 3. Results

### 3.1. PTBP1 Is High Expression in GBM and Correlates with Patient Age, WHO Grade, IDH Status, and Ki-67 Index

**Figure 1:**
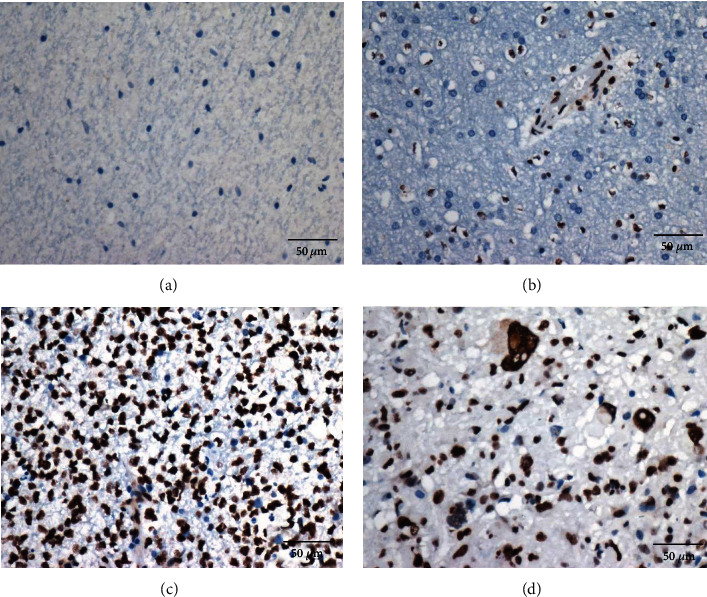
The PTBP1 protein expression. (a) No expression in normal brain tissues; (b) low expression in a WHO 2 diffuse astrocytoma case, and vascular endothelial cells were used as an internal positive control; (c) high expression in a WHO 3 anaplastic astrocytoma case; (d) high expression in a glioblastoma case (WHO 4).

**Figure 2:**
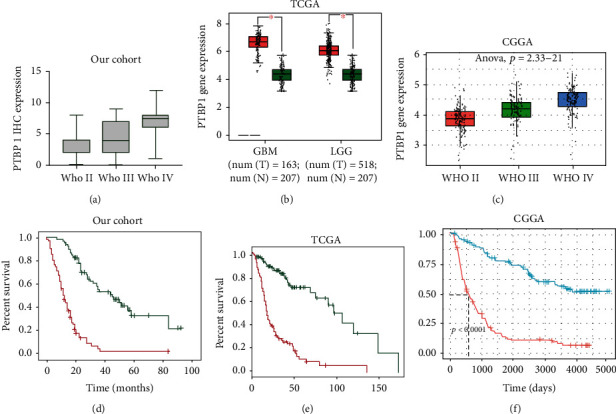
PTBP1 expression and KM survival curve. (a) PTBP1 protein expression in our cohort, PTBP1 mRNA expression in (b) TCGA and (c) CGGA. PTBP1 survival curve in (d) our cohort, (e) TCGA, and (f) CGGA.

The median PTBP1 expression level in malignant glioma (WHO 2-4) was used as the cut-off point to divide the patients into low-PTBP1 and high-PTBP1 expression groups. PTBP1 protein was highly expressed in glioma tissues (79/150, 52.7%), but no expression was detected in normal brain tissues (0/20, 0%) in our cohort (Figure 1). The expression of PTBP1 was significantly higher in GBM. The high expression rate of PTBP1 increased with WHO grade (4/30, 13.3% in WHO 2; 13/30,43.3% in WHO 3; and 62/90, 68.9% in WHO 4). PTBP1 expression was significantly higher in grade 4 than in either grades 2 (*P* < 0.001) or 3 (*P* < 0.001), and PTBP1 expression in grade 3 glioma was higher than that in grade 2 (*P* = 0.0038). The same results were found in TCGA and CGGA datasets (Figure 2). Furthermore, the public database Oncomine was used to further confirm these findings. The mRNA levels of PTBP1 are significantly increased in GBM compared with normal brain tissues in the Oncomine database (Table 1). Then, the clinicopathological information was investigated using chi-square test, and we found PTBP1 expression was associated with patient age (*P* = 0.036), WHO grade (*P* < 0.001), IDH status (*P* < 0.001), and Ki-67 (*P* = 0.002). However, the expression of PTBP1 was not significantly correlated with gender, tumor location, and KPS. Details are listed in Table 2.

### 3.2. High PTBP1 Expression Predicts Significantly Poor Prognosis, and It Is an Independent Prognostic Marker in Glioma

Kaplan-Meier survival curves and the log-rank test were employed to identify any associations between PTBP1 expression and OS. Patients in the low-PTBP1 expression group lived significantly longer compared with those in the high expression group in our cohort (*P* < 0.001) (Figure 2(d)). This data demonstrated that high expression of PTBP1 may be indicative of an unfavorable survival outcome. TCGA and CGGA datasets were also analyzed for confirmation of our findings, and the results also demonstrated that the group with a high expression of PTBP1 had a significantly worse outcome (*P* < 0.001) compared with the low expression group (Figures 2(e) and 2(f)). Univariate Cox regression analysis was conducted to analyze the genetic and clinical variables with respect to survival. Subsequently, potential prognostic factors associated with OS were evaluated through a multivariate Cox regression model. The results demonstrated that PTBP1 expression was an independent prognostic factor for OS in glioma (hazard ratio (HR), 4.901; 95% confidence interval (CI), 2.778-8.645; and *P* < 0.001) (Table 3).

### 3.3. Construction of Transcriptional Network of PTBP1 in Glioma

**Figure 3:**
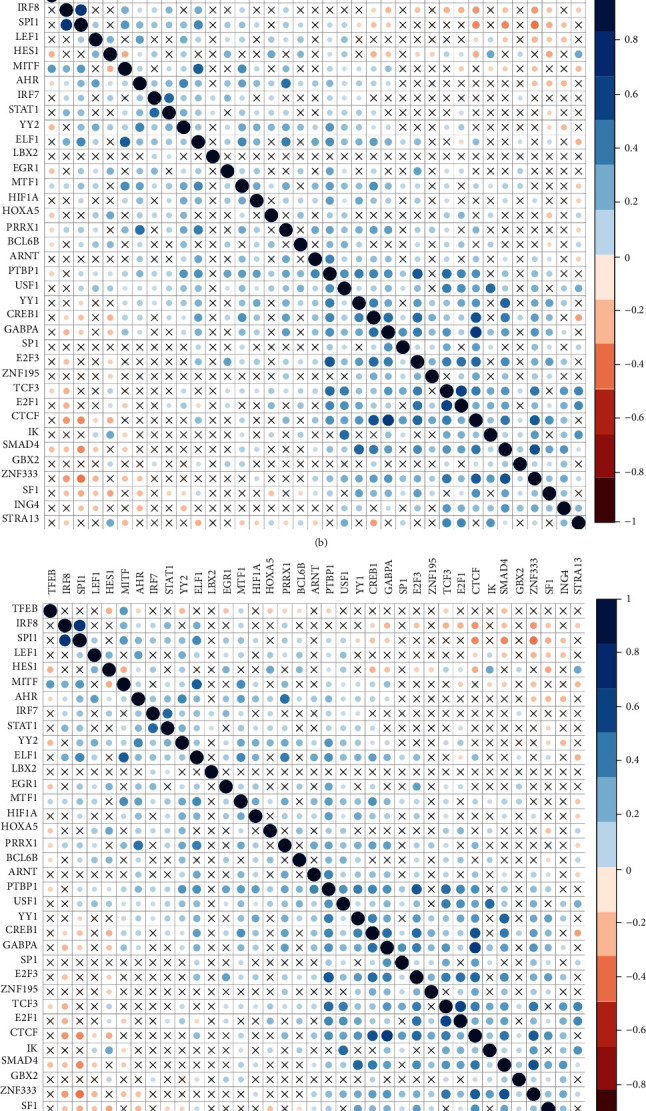
The transcriptional network of PTBP1 in glioma. (a) 126 predicted transcription factors (TFs) related to PTBP1 through the GCBI database; (b, c) the correlation between 37 TFs and PTBP1 in TCGA and CGGA databases; (d) TCGA TFs and CGGA TFs; (e) the correlation between TCF3 and PTBP1; (f) the expression of TCF3 in glioma and normal tissues; (g) the predicted upstream miRNAs of TCF3 and PTBP1; (h) the miR-137 and PTBP1 and TCF3.

To further explore the potential upstream mechanism of PTBP1 involved in glioma progression, we predicted 126 transcription factors (TFs) related to PTBP1 through the GCBI database and screened out 74 TFs using the Funrich software. Furthermore, 37 TFs were further verified to be highly expressed in gliomas through GEPIA tool. We further analyzed the correlation between 37 TFs and PTBP1 in TCGA and CGGA databases. Finally, we found that TCF3 showed highly positive correlation with PTBP1 in glioma and was significantly higher in glioma compares to normal tissues (*P* < 0.001, *r* = 0.7). Simultaneously, we predicted the upstream miRNAs of TCF3 using miRanda and Targetscan databases and compared them with the miRNA upstream of PTBP1. miR-137 was the only overlapping miRNA in our prediction system. Given these above results, we proposed that PTBP1 could be directly inhibited by miR-137 or indirectly inhibited by miR-137-mediated suppression of TCF3 (Figure 3).

### 3.4. Biological Enrichment Analysis of PTBP1 Downstream Pathway in Glioma

**Figure 4:**
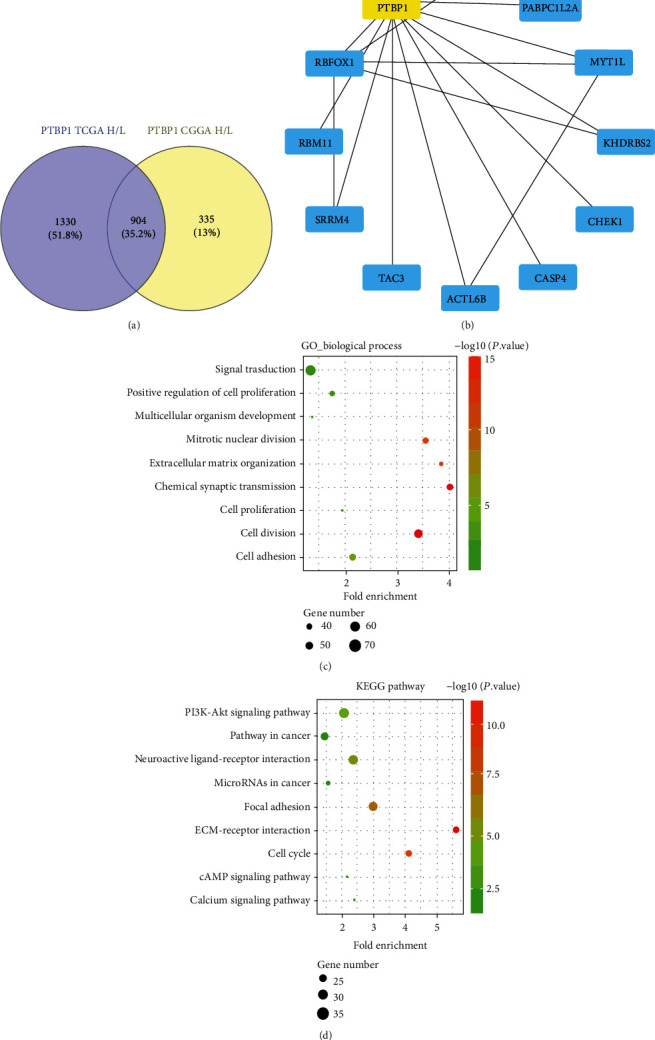
Biological enrichment analysis of PTBP1 downstream pathway in glioma. (a) The differentially expressed genes (DEGs) according to the expression level of PTBP1 in TCGA and CGGA databases; (b) 11 DEGs are directly related to PTBP1 by using the STRING database and the Cystoscope software; (c) the GO process of DEGs; (d) the KEGG pathway of DEGs.

To analyze the PTBP1 downstream pathway, we firstly screened out the differentially expressed genes (DEGs) according to the expression level of PTBP1 in TCGA and CGGA databases. In each database, we group samples based on extreme PTBP1 expression. The DESeq R package was used to screen DEGs, cut-off criteria set as |logFC| > 1.5, and *P* < 0.05. We identified 904 DEGs overlapping among TCGA and CGGA databases through a Venn diagram (Figure 4(a)). Among them, 11 DEGs are directly related to PTBP1 by using the STRING database and the Cystoscope software (Figure 4(b)). Moreover, the potential roles of PTBP1 and 904 DEGs were further explored by using the DAVID database. Gene ontology analysis showed that they were mainly members of the ubiquitin ligase complex and associated with posttranslational protein modification, which participated in the regulation of ubiquitin protein ligase binding (Figure 4(c)). Furthermore, the potential signaling pathways associated with PTBP1 and 904 DEGs were predicted using the KEGG pathway database. Results showed that they closely related to PI3K-Akt signaling pathway (Figure 4(d)).

## 4. Discussion

Malignant gliomas have been always an important cause of death in adults and children in brain tumors. The multiple genetic changes caused the formation, progression, invasion, and maintenance of these highly malignant tumors. Genomic methods have been used in several studies to determine the underlying cause of this cancer. In fact, as part of The Cancer Genome Atlas project (TCGA), GBM was one of the earliest tumor types to be included in the study [3]. The determination of glioma molecular features is accompanied by the beginning of stratified treatment strategies [4].

With the continued development of methods for detecting whole-genome RNA splicing, there is a greater understanding of the role of RNA processing in the creation of genetic diversity and the regulation of cellular functions. Researches using high-throughput sequencing methods have shown that more than 90% of protein-coding genes produce alternative mRNAs [5, 6]. Selective RNA splicing changes the function of the protein by changing the protein domain. Protein-coding genes can generate noncoding circular RNAs (circRNAs) by back-splicing. Due to their stable structures that are not easily degraded, some studies have pointed out that circRNAs can be used as serum biomarkers to assess the prognosis and diagnosis of glioma [7]. More importantly, misregulation may lead to the activation of oncogenes or the inactivation of tumor suppressor genes, leading to tumorigenesis [8, 9].

Human polypyrimidine bundle binding protein 1 (PTBP1) is a member of the hnRNP family of RNA-binding protein that moves between the nucleus and the cytoplasm and regulates the many RNA posttranscriptional processes [10]. It has been suggested that PTB protein recognizes specific pyrimidine-rich sequence in the 3′ splice site and binds to its cognate site results in inhibition of nearby splice sites, thereby protecting the RNA exon from nonspecific splicing [11, 12]. There is increasing evidence that PTBP1 can involve in the alternative splicing of multiple genes in glioma and play a promoting role in glioblastoma tumorigenesis. The results of Izaguirre et al. [13] have supported that PTBP1 can modulate the alternative splicing of USP5. Ferrarese et al. [9] have indicated that PTBP1 augments EGFR signaling through ANXA7 splicing to promote tumor angiogenesis. Yang et al. [14] have revealed PTBP1 can activate the ADAR1 p110 isoform through an IRES-like element to maintain glioma formation and regulate the glioma cell proliferation. Aldave et al. [15] have demonstrated that BAF45d splicing is mediated by PTBP1, and BAF45d transcription regulates PTBP1 in turn, revealing an interaction between RNA splicing regulation and transcription. Barbagallo et al. [12] demonstrated glioma cells can be positively regulated to migration by splicing factors SRSF1/SRSF3/PTBP1. There is no study on the expression of PTBP1 in Chinese glioma population. In this study, we detected the expression of PTBP1 in 150 cases of adult malignant gliomas by immunohistochemistry and evaluated its relationship with clinicopathological parameters by chi-square test. We found the PTBP1 expression was increased with WHO grade both in our cohort and CGGA/TCGA datasets, and the expression was highest in glioblastoma. McCutcheon et al. [16] studied the expression of PTBP1 in 17 different types of brain tumors. They have demonstrated the PTBP1 expression in the brain tissues, including 2 cases of normal brain and 17 cases of different types of brain tumors. However, we found no PTBP1 expression in 20 cases of normal brain tissues in our cohort, except vascular endothelium stained strongly as an internal control. In our tumor samples, intense positive nuclear staining was observed in high-grade glioma, particularly in GBM, indicating strong upregulation of PTBP1 expression in tumor cells of glial suggests involvement of this protein in cellular malignant transformation. PTBP1 affects splicing of RNAs critical to cellular transformation, and proliferation could be the underlying mechanism. And this is consistent with Zhu et al.'s results [17]. They also reported PTBP1 overexpressed in a wide variety of glia-derived tumors and aberrant RNA splicing may play an important role either in glial cell transformation or in progression of lower-grade astrocytic neoplasms to glioblastoma. Furthermore, we used bioinformatics to predict the potential upstream mechanisms and downstream pathways of PTBP1's involvement in glioma progression. We have explored miR-137-TCF3-PTBP1 regulatory interactions by analyzing the gene expression from TCGA and CGGA databases. And further experimental confirmation is needed. Importantly, we found PTBP1 was closely related to prognosis in glioma, with high expression indicating poor prognosis both in our study and CGGA and TCGA datasets. A possible explanation may be that PTBP1 is mainly expressed in GBM, which is the most malignant type. However, PTBP1 was still an independent prognostic factor in the multivariate Cox analysis, which also indicated its important prognostic significance.

In conclusion, these results demonstrate PTBP1 serve as a promising prognostic biomarker in glioma, and patients with high-PTBP1 expression need more aggressive treatment. PTBP1 may be a potential therapeutic target for glioma, particularly for GBM.

## Figures and Tables

**Table 1:** PTBP1 expression in the Oncomine glioma database.

Upregulation of PTBP1 in glioblastoma	*P* value	Fold change	Database
Glioblastoma (542) vs. normal (10)	1.32*E*-10	5.091	TCGA brain
Glioblastoma (80) vs. normal (4)	3.31*E*-6	3.198	Murat brain
Glioblastoma (81) vs. normal (23)	2.20*E*-21	3.049	Sun brain

**Table 2:** Clinical-pathological characteristics of the patients and PTBP1 expression.

	No. of cases	PTBP1		*P*
		Low	High	
Age				0.036
≤55	113	59	54	
>55	37	12	25	
Gender				0.129
Male	94	40	54	
Female	56	31	25	
WHO grade				<0.001^∗^
2-3	70	53	17	
4	80	18	62	
Location				0.624
Supratentorial	147	70	77	
Subtentorial	3	1	2	
IDH				<0.001^∗^
Wildtype	99	32	67	
Mutated	51	39	12	
KPS				0.233
≤70	61	28	33	
>70	89	43	46	
Ki-67				0.002
≤10%	22	17	5	
>10%	128	54	74	

^∗^ *P* < 0.05.

**Table 3:** Univariate and multivariate analyses for overall survivals.

Variable	Univariate analysis		Multivariate analysis	
	Hazard ratio (95% CI)	*P* value	Hazard ratio (95% CI)	*P* value
Gender: male	1.231 (0.880~1.983)	0.179		
Age: >55	2.116 (1.397~3.207)	<0.001^∗^	1.464 (0.950-2.257)	0.084
Location: supratentorial	0.546 (0.173~1.729)	0.304		
WHO grade: WHO 4	1.845 (1.411~2.414)	<0.001^∗^	0.823 (0.558-1.213)	0.325
IDH: mutated	0.308 (0.191~0.494)	<0.001^∗^	0.613 (0.338-1.113)	0.108
KPS: >70	0.721 (0.488~1.066)	0.102		
Ki67: >10%	2.225 (1.182~4.187)	0.013^∗^	1.624 (0.802-3.289)	0.178
PTBP1: high	5.807 (3.732~9.036)	<0.001^∗^	4.901 (2.778-8.645)	<0.001^∗^

^∗^ *P* < 0.05.

## Data Availability

The analyzed datasets generated during the study are available from the corresponding author on reasonable request.
